# Timelines, targets, and transitions in cardiovascular care

**DOI:** 10.1007/s12471-025-01984-x

**Published:** 2025-08-04

**Authors:** Pim van der Harst

**Affiliations:** Department of Cardiology, Division of Heart and Lungs, University Medical Centre Utrecht, Utrecht, The Netherlands

This issue of the *Netherlands Heart Journal* presents 5 contributions that reflect current efforts to strengthen cardiovascular care in the Netherlands through system-level insight, protocol-based interventions, and new care models.

Camaro et al. provide a nationwide analysis of invasive strategies in NSTEMI. While most patients undergo coronary angiography within guideline-recommended timelines, the time to PCI still varies substantially by hospital type. These data help contextualise the 2023 ESC guidelines and their national endorsement [1], and highlight how institutional structures influence real-world delivery.

Van Trier et al. evaluate protocol-led LDL‑C management following myocardial infarction, showing superior target attainment at both 6 months and 1 year compared to routine care. Their findings expand on earlier reports of structured lipid-lowering (Khader et al. [2]) and legacy effects (van der Brug et al. [3]), reinforcing the role of sustained, organised secondary prevention strategies.

Two articles focus on atrial fibrillation (AF). Middeldorp et al. show that healthcare utilisation is driven more by multimorbidity than by age, supporting personalised, integrated care. Hengstman et al. report on nurse-led home cardioversion, showing feasibility in a selected population and opening the door to decentralised AF management.

Accord et al. describe the first Dutch experience with combined heart—liver transplantation (CHLT) in two adult patients. One involved adult congenital heart disease—a scenario in which transplantation remains particularly rare, even in children [4].

We thank all authors for their work and invite readers to engage with the thoughtful, practice-oriented content in this issue.
